# Response of Rhizosphere Microenvironment of Mulberry (*Morus alba* L.) to Different Cultivars

**DOI:** 10.3390/microorganisms13092157

**Published:** 2025-09-16

**Authors:** Chuanjie Chen, Haiyang Zhang, Xiaoyan Liang, Meng Li, Yinyu Gu

**Affiliations:** 1 Shandong Institute of Sericulture, Shandong Academy of Agricultural Sciences, Yantai 265503, China; 2 Shandong Engineering Research Center of Functional Crop Germplasm Innovation and Cultivation Utilization, Yantai 265503, China; 3 Yantai Engineering Research Center of Plant Stem Cell Targeted Breeding, Yantai 265503, China; 4 Yantai Key Laboratory of Evaluation and Utilization of Silkworm Functional Substances, Yantai 265503, China

**Keywords:** response, rhizosphere microenvironment, mulberry, cultivar

## Abstract

Soil microbiomes have a crucial role in mulberry development; however, the correlation between the mulberry genotype and rhizosphere microenvironment has not been explored. The rhizosphere microbial community structure and function of rizhosphere bacteria and fungi in five mulberry cultivars and their interaction with soil chemical properties and agronomic traits were analyzed using Illumina-based sequencing. We demonstrated that the composition, structure, and assembly processes of rhizosphere bacteria and fungi exhibited significant differences among mulberry cultivars, and their response to soil chemical traits and leaf yield also varies. The correlations in the bacterial communities were more complex than in the fungal communities among the five cultivars. During the assembly process, bacteria were more stable than fungi. *Penicillium* and *Phytophthora* showed a positive correlation with leaf yield and were significantly enriched in the Canghai 12 rhizosphere soil, which exhibited the highest leaf production. *Bacillus* was a bacterium that showed a significant positive correlation with leaf yield. The saprotrophs exhibited the largest guild in terms of operational taxonomic unit richness. This research indicated that the mulberry genotype is one of the dominant factors in rhizosphere microorganism recruitment and assembly. These findings provide new insights into the complex microbial community soil–plant interaction and probiotic screening.

## 1. Introduction

*Morus alba* L. (mulberry), a significant species within the Moraceae family, has been widely historically cultivated worldwide and is predominantly found in the subtropical regions of Asia as well as in various countries in Africa, North America, and Europe [1]. *M. alba* has multiple applications. It has edible and medicinal properties. Moreover, it is a primary food source for silkworms (*Bombyx mori* L.), and silk in turn has various applications, such as in clothing, household materials, and cosmetics [2,3]. Different mulberry species contain diverse extracts that demonstrate considerable therapeutic potential in treating various diseases [4]. The usefulness to human health in addition to its nutrition-rich leaves and fruits makes the plant suitable to be placed in the category of functional foods [5]. Furthermore, mulberry trees exhibit potential for the bioremediation of heavy metal-contaminated soil and polluted atmosphere, contributing to environmental sustainability [6,7,8]. Although every part of the mulberry tree can be utilized [9], the leaves remain the most widely used part [10]. Due to differences in genotypes and environments, leaf yield differ greatly among different mulberry cultivars. Therefore, improving mulberry leaf yield through breeding, cultivation management, and environmental improvement has been a key research area for scientists.

The soil nutrients are absorbed by the plant through the root system. The rhizosphere serves as the central zone where interactions among plant roots, soil, microorganisms, and the surrounding environment take place [11]. Root exudates interact with soil microorganisms in the rhizosphere, contributing to the formation of a diverse and dynamic microbial community. These interactions play a crucial role in mitigating issues, such as abiotic stress, plant diseases, and pest infestations [12]. Rhizosphere microorganisms are to be recognized as a beneficial, sustainable, and economic approach for agriculture [12]. The plant growth-promoting rhizobacteria (PGPR) in the rhizosphere can convert nitrogen, phosphorus, zinc, etc., into forms that plants can readily absorb. The PGPR improve soil fertility and its functions and enhance plant growth by producing plant hormones, such as auxins and cytokinins [13]. Bacteria are the major component of plant microbiome, followed by fungi [11]. There are some reports that beneficial microorganisms play a crucial role in maintaining the ecological balance within the rhizosphere of a mulberry plant. Vesicular-Arbuscular Mycorrhizal (VAM) fungi can enhance the rhizosphere microbial community structure and function, thereby improving resistance to pathogenic infections. The synergistic applications of antagonistic fungi with VAM and effective microorganisms have been shown to effectively prevent and manage root rot disease in mulberry [14]. Moreover, plant growth-promoting fungi has been shown to markedly improve mulberry tree growth, enhance drought tolerance, and consequently contribute to increased mulberry fruit and leaf yield [15]. Therefore, rhizosphere microorganisms of a mulberry tree can affect the crop yield and subsequently influence the economic value of the crop.

Plant genotypes are also closely linked to rhizosphere microorganisms. Rhizosphere microbial communities exhibit differences across various *M. alba* genotypes. Resistant plant genotypes have more stable bacterial and fungal communities than sensitive plant genotypes [16]. Different mulberry genotypes can influence the composition, diversity, and abundance of soil microbial communities, thereby affecting the resistance to soil-borne diseases. A reduction in beneficial fungal species or overall fungal diversity may compromise the plant’s resistance to soil-borne diseases (e.g., Mulberry fruit sclerotiniosis) [17]. The rhizosphere fungal diversity is relatively low in fruit rot-susceptible mulberry genotypes [18]. Previous studies have explored the correlation between rhizosphere microorganisms of different mulberry genotypes and disease resistance. However, limited research has been conducted on the correlation between soil nutrients and leaf yield.

This study aims to investigate the differences in rhizosphere microbial communities among various *M. alba* genotypes as well as the correlation between these microorganisms and soil physicochemical properties and agronomic traits of mulberry under identical natural conditions. The objectives of this study were as follows: (1) Determine the characteristics of bacteria and fungi community diversity changes among mulberry cultivars. (2) Characterize the assembly features of rhizosphere bacteria and fungi associated with mulberry cultivars. (3) Illustrate the correlation between mulberry rhizosphere microbial communities, mulberry rhizosphere soil chemical properties, and mulberry leaf yield. (4) Screen microorganisms beneficial to mulberry growth.

## 2. Materials and Methods

### 2.1. Sample Collection

Mulberry cultivars, “Husang 32” (H32), “Canghai 12” (CH12), “Canghai 7” (CH7), “Jinxuan 10” (JX10), and “Shansang 908” (SS908), were cultivated in the experimental field of Shandong Sericulture Research Institute (37°08′25.44″ N, 121°08′33.98″ E) in Yantai, Shandong Province, China, in March 2019. The JX10 and SS908 cultivars were bred by Shanxi province, CH7 and CH12 cultivars were bred by Shandong province, and H32 was a control cultivar. All cultivars were propagated uniformly by grafting, and were cultivated according to Table S1.

The mean air temperature was 14 °C, average annual rainfall was approximately 700 mm, with a frost-free period of more than 210 d, and an annual sunshine duration of more than 2100 h. In May 2024, soil samples were collected from three healthy looking, medium-growth plants randomly selected from each block (three lines) of each cultivar, and were pooled to form one replicate. Each cultivar had three replicates, resulting in a total of 15 pooled soil samples for testing. The rhizosphere soil was sampled from a depth of 0–20 cm. Each sample was divided into two sections after cleaning: one was immediately quick-freezed in liquid nitrogen and sent to Majorbio Bio-Pharm Technology Co., Ltd. (Shanghai, China) for high-throughput sequencing, and the other section was air-dried and sieved for the measurement of soil chemical properties. The agronomic traits were investigated in June, including leaf yield per plant and frozen shoot rate.

### 2.2. Soil Chemical Analysis

Soil pH values were determined using a Shanghai Lei magnetic multi-parameter water quality analyzer DZS-708 (INESA, Shanghai, China). Total phosphate (TP) and available phosphorous (AP) content was measured using ICP-MS after microwave digestion (MARS5, CEM, Matthews, NC, USA) (0.1 g sample + 6 mL concentrated nitric acid). Alkaline nitrogen (AN) and total nitrogen (TN) contents were determined using an elemental analyzer (FLASH-2000, Thermo Scientific, Waltham, MA, USA). Soil organic matter (SOM) was quantitated using the potassium dichromate oxidation method using an SSM-5000A (Shimadzu, Kyoto, Japan) carbon analyzer. Soil total potassium (TK) and available potassium (AK) were extracted with 1 mol/L ammonium acetate (pH 7.0) and determined using an atomic absorption spectrophotometer AA4590 (Aupos Scientific, Duesseldorf, Germany).

### 2.3. Soil Microbial Community Analysis

Microbial genomic DNA was extracted from rhizosphere soil samples using the E.Z.N.A.^®^ soil DNA kit (Omega Bio-Tek, Norcross, GA, USA) and subsequently checked for concentration and purity using a NanoDrop 2000 UV-Vis spectrophotometer (Thermo Scientific, CA, USA). The V3-V4 region of the bacterial 16S rRNA gene was amplified using the primers, 338F (5′-ACTCCTACGGGAGGCAGCAG-3′) and 806R (5′-GGACTACHVGGGTATCTAAT-3′). The primers, namely, ITS1F (5′-CTTGGTCATTTAGAGGAAGTAA-3′) and ITS2R (5′-GCTGCGTTCTTCATCGATGC-3′) were used to amplify the internal transcribed spacer (ITS). The extracted PCR products were quantified using a Quantus™ fluorometer (Promega Corporation, Madison, WI, USA) after purification. The purified amplicons were pooled and paired-end sequenced by Majorbio Bio-Pharm Technology Co. Ltd. (Shanghai, China) using an Illumina MiSeq PE300 platform (Illumina Inc., San Diego, CA, USA). More details can be found in the supplementary Table S2. The complete sequences generated in this study are available in the NCBI SRA database under accession number SRA data: PRJNA1302645.

### 2.4. Data Analysis

Calculations were performed using Microsoft Excel, and the statistical analyses were performed using DPS Statistics 18.10 software (http://www.dpsw.cn). All analyses were performed on the Majorbio Cloud Platform (www.majorbio.com). α-diversity was calculated using Mothur software (v.1.30.2). R script (v.3.3.1) was used to analyze and generate Venn, Bar, and redundancy analysis (RDA) diagrams. β-diversities were visualized using principal coordinates analysis (PCoA) based on the distance matrix, with bray_curtis. The normalized stochasticity ratio (NST) was determined with NST packages (3.1.10) in R packages (version 3.3.1), the niche breadth of bacterial and fungal communities was measured by spaa (version 0.2.2) of R packages (version 3.3.1.), and community sensitivity was measured using python (version 2.7.10). Linear discriminant analysis effect size (LEfSe) was performed through software (http://galaxy.biobakery.org/). A network analysis was performed to explore the interaction complexity among the microbial taxa using Networkx (version 1.11). Finally, the potential functions of bacterial communities were predicted using the phylogenetic investigation of communities by reconstruction of unobserved states PICRUSt2 (http://huttenhower.sph.harvard.edu/galaxy), and fungi were classified into functional guilds by FUNGuild (http://www.funguild.org/). Data are presented as the means and standard errors.

## 3. Results

### 3.1. Agronomic Traits and Soil Chemical Properties

**Figure 1:**
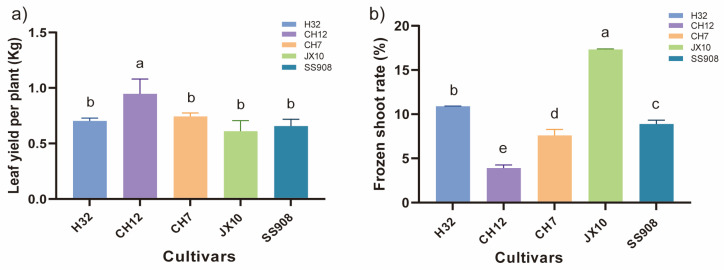
Leaf yield and frozen shoot rate. H32: Mulberry cultivar “Husang 32”; CH12: Mulberry cultivar “Canghai 12”; CH7: Mulberry cultivar “Canghai 7”; JX10: Mulberry cultivar “Jinxuan 10”; SS908: Mulberry cultivar “Shansang 908″. Error bars represent standard errors of the means (n = 3). Different lowercase letters indicate significant difference among treatments (*p* < 0.05). (**a**) Leaf yield per plant. (**b**) Frozen shoot rate.

The leaf yield per plant in the CH12 cultivar was the highest among all the five cultivars, with no significant differences among the other four cultivars and the lowest yield in the JX10 cultivar (Figure 1a). With respect to the frozen shoot rate, the JX10 cultivar exhibited the highest potential, followed by the H32 cultivar, and the CH12 cultivar presented the lowest potential (Figure 1b).

The CH7 cultivar exhibited the highest values for all the indicators. Specifically, no significant differences in TN, TP, and SOM were observed among the other four cultivars. The H32, and CH7 cultivars had significantly higher TK levels than the other three cultivars. In terms of the AN content, the CH12 cultivar ranked second only to the CH7 cultivar, exhibiting significantly higher AN values than the other three cultivars. The AP levels were the lowest in the JX10 cultivar than the other four cultivars. The CH7 cultivar exhibited the highest AK value, followed by the CH12 and SS908 cultivars, whereas the JX10 and H32 cultivars had the lowest AK value. Finally, the CH7 cultivar exhibited the highest pH, whereas the SS908 cultivar had the lowest (Table 1).

### 3.2. α-Diversity Index Analysis and Composition

In all the 15 samples, the rarefaction curves tended to approach the saturation plateau (Supplementary Figures S1 and S2). Combined with the estimated coverage values (Supplementary Tables S3 and S4), this indicated that the data were sufficiently large enough to capture most of the bacterial and fungal diversity in the samples. The richness of bacteria was the highest in the H32 cultivar, which was significantly higher than the CH7, JX10, and SS908 cultivars. However, the highest fungal richness was possessed by the CH7 cultivar, whereas the SS908 cultivar had the lowest. No significant differences in bacterial or fungal diversity were observed among different cultivars tested.

**Figure 2:**
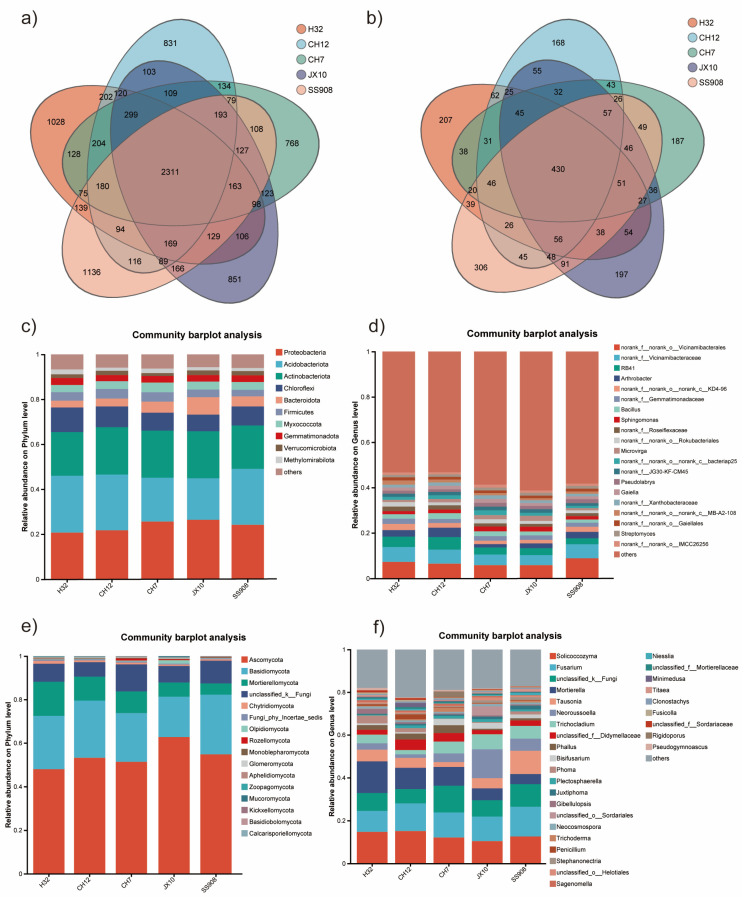
Composition of microorganisms. (**a**) Venn diagram of bacteria based on OTUs. (**b**) Venn diagram of fungi based on OTUs. (**c**) Bar of bacteria based on phylum. (**d**) Bar of bacteria based on genus. (**e**) Bar of fungi based on phylum. (**f**) Bar of fungi based on genus.

The number of common and unique bacterial operational taxonomic units (OTUs) in the different cultivars is shown in Venn diagrams (Figure 2a). A total of 10,378 OTUs were detected across all libraries with 2311 OTUs common to different cultivars, and the SS908 cultivar harbored the highest unique OTUs (1136), followed by the H32 cultivar (1028), and then the JX10, CH12, and CH7 cultivars. The fungal OTUs obtained were far lower than those of bacteria (Figure 2b). The five mulberry cultivars shared 430 OTUs of the total 2581 OTUs, with the highest unique OTUs in the SS908 cultivar (306), and the lowest unique OTUs in the CH12 cultivar (168), similar to the bacteria.

All bacterial OTUs were classified into 43 phyla, 133 classes, 322 orders, 495 families, 930 genera, and 2,211 species. The dominant bacterial phyla were Proteobacteria, Acidobacteriota, and Actinobacteriota, accounting for more than 60% of the total bacterial community (Figure 2c). Over 15% percent of bacterial relative abundance was occupied by *g__norank_f__norank_o__Vicinamibacterales*, *g__norank_f__Vicinamibacteraceae*, *RB41*, and *Arthrobacter* in all the cultivars (Figure 2d). All fungal OTUs were classified into 16 phyla, 50 classes, 119 orders, 267 families, 502 genera, and 709 species. The dominant fungi phyla in all cultivars were Ascomycota, Basidiomycota, and Mortierellomycota, which harbored more than 80% of the total fungal community (Figure 2e). The top two fungal genera were *Solicoccozyma* and *Fusarium*, which occupied over 20% across five cultivars (Figure 2f).

### 3.3. β-Diversity

**Figure 3:**
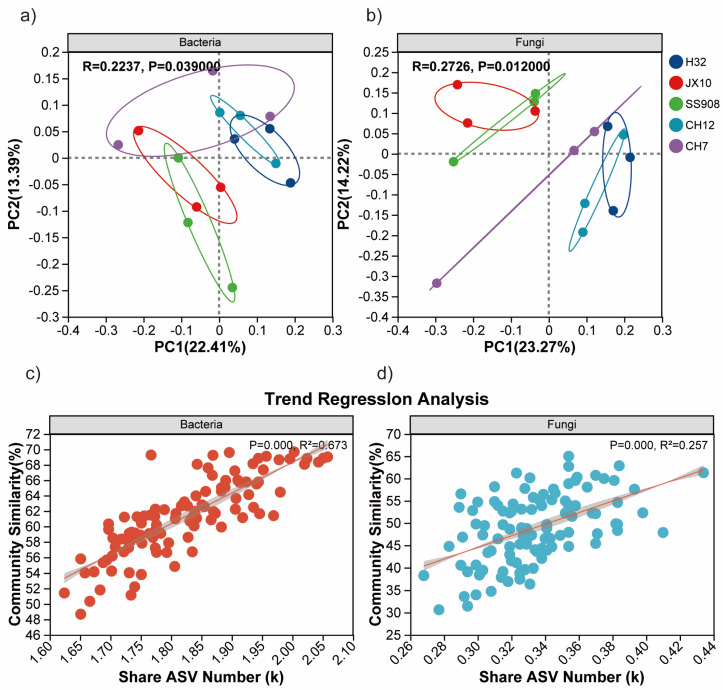
(**a**) PCoA of bacteria based on genus. (**b**) PCoA of fungi based on genus. (**c**) NST of bacteria based on genus. (**d**) Community similarity in bacteria based on Bray–Curtis index.

To further identify the microbial population associated with different cultivars, PCoA was conducted to examine the differences in rhizosphere soil bacterial and fungal communities (Figure 3a,b). For bacteria, PCoA identified two principal component factors related to the percent abundance of the cultivars, explaining 22.38% (PC1) and 13.41% (PC2) of the variation. The clustering of different cultivars indicated that the community structures were similar between the H32 and CH12 cultivars. Likewise, the community structures were similar between the JX10 and SS908 cultivars. However, the community structures in the CH7 cultivar were different from the other cultivars. In contrast, there were significant differences in the community structures of the H32-CH12 and JX10-SS908 cultivars. With respect to fungi, two principal components explaining 23.24% (PC1) and 14.15% (PC2) of the variation in fungal abundance among samples were identified. Similarly to bacteria, the H32 and CH12 cultivars clustered together and the JX10 and SS908 cultivars clustered together, whereas the CH7 cultivar clustered separately.

### 3.4. Mechanisms of Bacterial and Microbial Community Assembly

We further compared the associations between the shared species numbers and the community similarities based on the Bray–Curtis analysis (Figure 3c,d). Significant linear correlations were observed between the shared species numbers and the community similarities in bacterial and fungal communities in all the cultivars. Among all the cultivars, the explained ratio of shared species numbers to the bacterial similarity (67.3%) was higher than that observed in case of fungal similarity (25.7%). These results indicated that species abundance distribution influenced the assembly of bacteria and fungi communities in cultivars.

**Figure 4:**
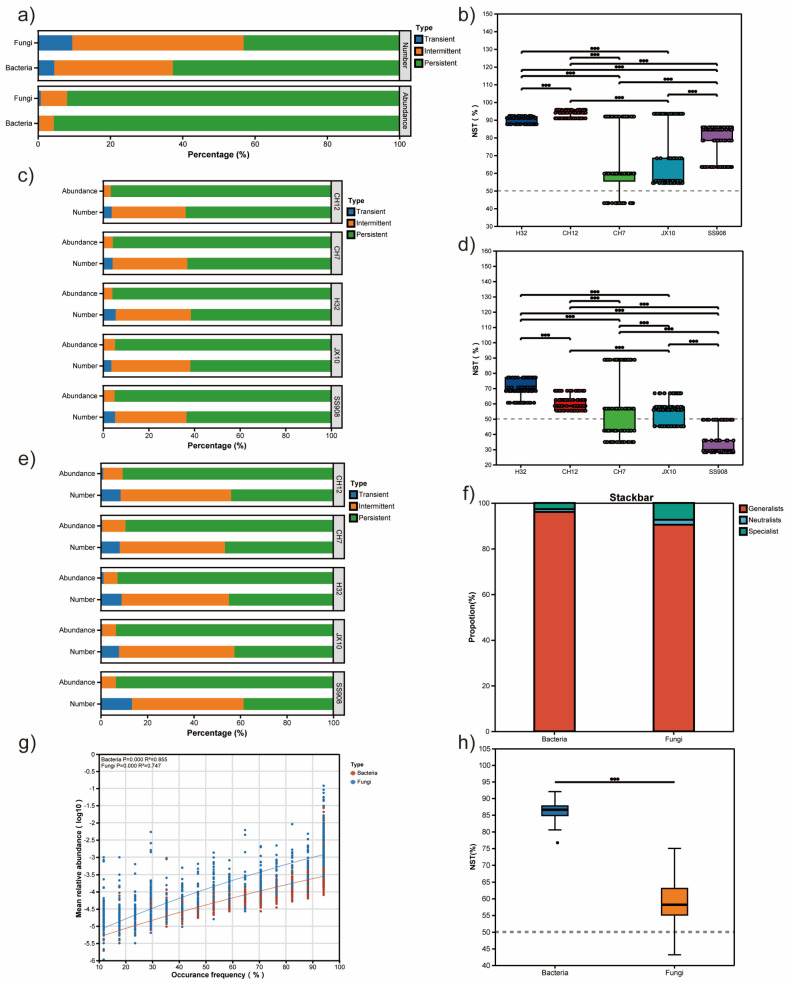
(**a**) Distribution of species in ecological communities. (**b**) NST of bacteria based on genus. (**c**) Distribution of bacterial species among different cultivars. (**d**) NST of fungi based on genus. (**e**) Distribution of fungal species among different cultivars. (**f**) Proportion of ecological bacterial and fungal community. (**g**) Mean relative abundance of bacteria and fungi. (**h**) NST of bacteria and fungi. NST: normalized stochasticity ratio. Note: “●●●” indicates a significant difference at *p* < 0.001.

The NST was calculated to quantify the roles of deterministic and stochastic processes of bacterial and fungal communities under different cultivars (Figure 4b,d,h). The NST values were above the 50% threshold for bacterial communities in the H32, CH12, CH7, JX10, and SS908 cultivars, with an average of 90.37%, 94.30%, 62.96%, 66.57%, and 79.06%, respectively, indicating that stochastic processes dominated the assembly of bacterial communities. Likewise, in terms of fungi, stochastic processes dominated in assembling the H32, CH12, CH7, and JX10 cultivar fungal communities with an average of 70.31%, 62.58%, 58.62%, and 55.25%, respectively. However, unusually, the SS908 cultivar demonstrated an NST value, which was less than 50%, indicating that deterministic process dominated the assembly of fungal communities. The NST value of bacteria was higher than that of fungi.

Bacteria and fungi were classified into persistent, intermediate, and transient species, and the differences in their numbers and abundances were analyzed. The most abundant bacterial and fungal species were persistent species, accounting for 95.57% and 91.93%, respectively. However, unlike the largest persistent species, the number of bacteria was 62.67%, the largest fungal species number was intermittent species (47.38%), followed by persistent species (43.07%) (Figure 4a,g). For bacteria, the pattern was consistent in the five cultivars (Figure 4c), whereas for fungi, only the CH7 cultivar exhibited persistent species occupying the highest number, with other cultivars being presented with intermittent species ranked first (Figure 4e).

**Figure 5:**
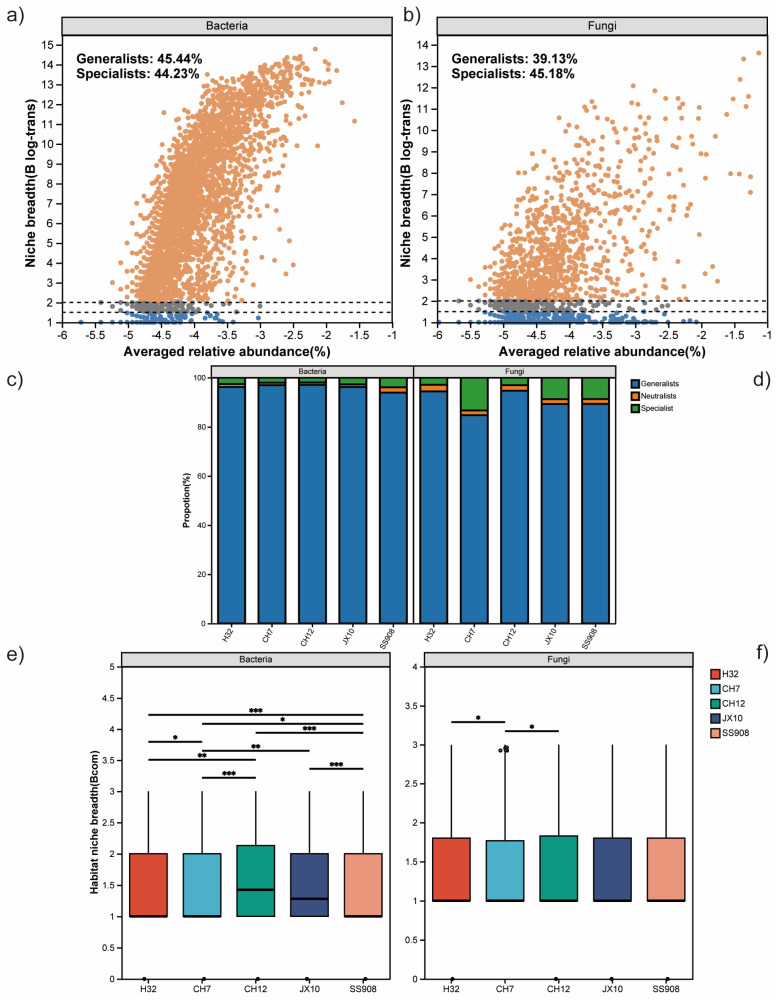
(**a**) Niche width scatter plot of bacteria. (**b**) Niche width scatter plot of fungi. (**c**) Proportion of ecological community of bacteria. (**d**) Proportion of ecological community of fungi. (**e**) Niche width of bacteria. (**f**) Niche width of fungi. Note: *** indicates a significant difference at *p* < 0.001, ** indicates a significant difference at *p* < 0.01, and * indicates a significant difference at *p* < 0.05.

In addition, the bacterial and fungal communities were further divided into generalists and specialists (Figure 5).The bacterial community had a greater proportion of generalists and a lower proportion of specialists than the fungal community (Figure 4f). Furthermore, the bacterial community had a higher niche width and overlap than the fungal community (Figure S3). For bacteria, the CH12 cultivar possessed the highest generalists, and the SS908 cultivar had the lowest generalists. With respect to fungi, the CH12 cultivar harbored the highest generalists, whereas the CH7 cultivar had the lowest generalists (Figure 5c,d). The ecological niche width was consistent with the above pattern (Figure 5e,f).

### 3.5. Difference Analysis

**Figure 6:**
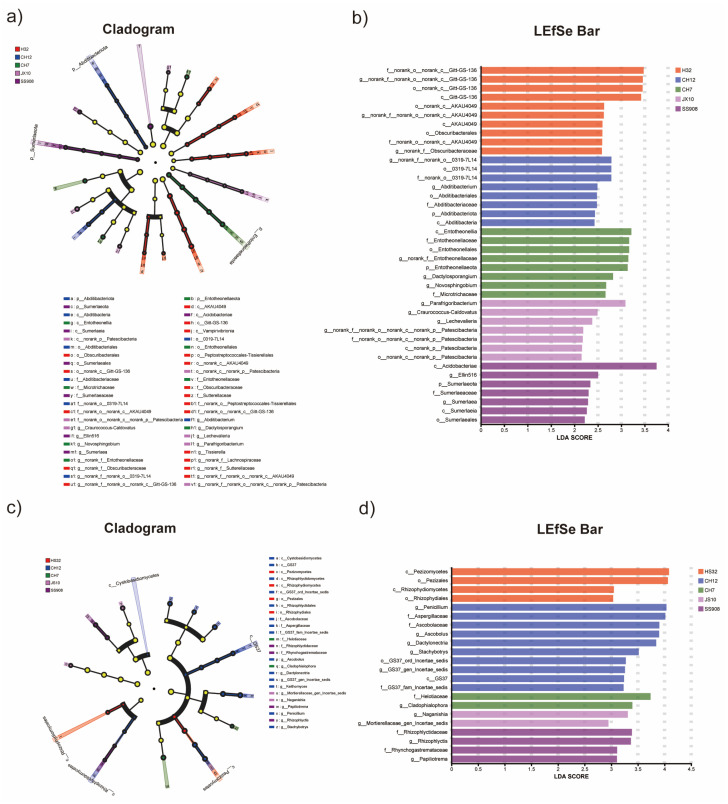
(**a**) LEfSe of bacteria. (**b**) LDA of bacteria. (**c**) LEfSe of fungi. (**d**) LDA of fungi.

LEfSe was conducted to determine significantly different taxa in different cultivars (Figure 4). With respect to bacteria, the H32 cultivar enriched two classes, three orders, two families, and three genera; the CH12 cultivar enriched one phylum, one class, two orders, two families, and two genera; the CH7 cultivar enriched one phylum, one class, one order, two families, and three genera; the JX10 cultivar enriched one class, one order, one family, and two genera; the SS908 cultivar enriched one phylum, one class, one order, one family, and two genera (Figure 6a,b). In terms of fungi, the H32 cultivar enriched two classes and two orders; the CH12 cultivar enriched one class, one order, three families, and five genera; the CH7 cultivar enriched one family and one genus; the JX10 cultivar enriched two genera; and the SS908 cultivar enriched one family and two genera (Figure 6c,d).

**Figure 7:**
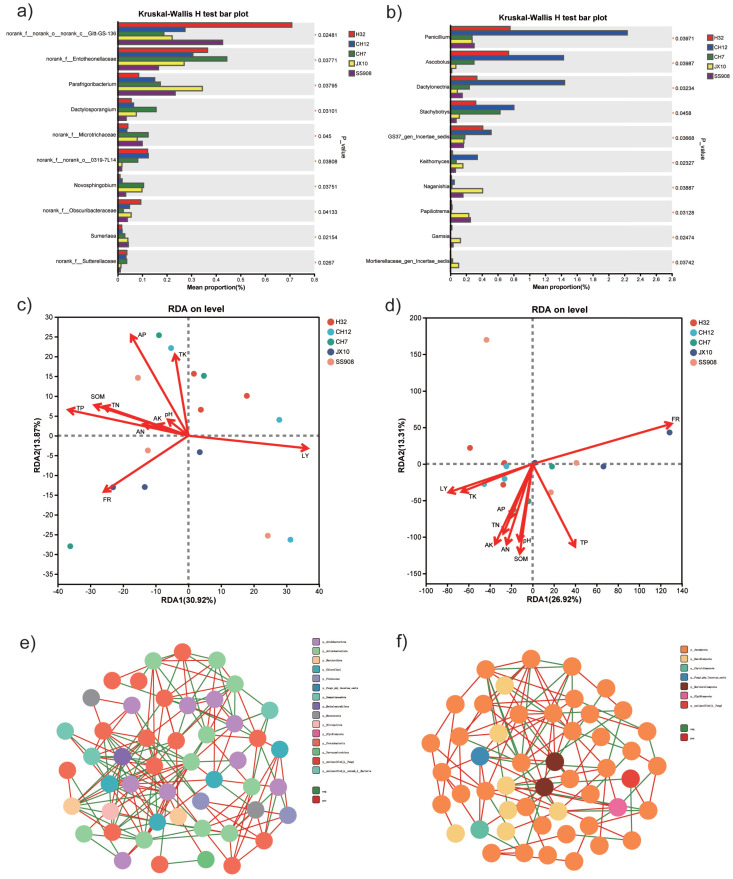
(**a**) Different bacterial genera. (**b**) Different fungal genera. (**c**) RDA of bacteria. (**d**) RDA of fungi. The *x*-axis represents the mean proportion of the genus and the *y*-axis indicates the top 10 dominant bacterial genera. (**e**) Correlation network analysis of bacteria among five cultivars. (**f**) Correlation network analysis of fungi among five cultivars. LY represents leaf yield, FR represents frozen shoot rate. Note: * indicates a significant difference at *p* < 0.05.

In addition, the relative abundance of core bacterial genera was compared, and the top 10 genera are shown in Figure 7a. The relative frequencies of *norank_f_norank_o_norank_c_Gitt-GS-136* were the highest in the H32 cultivar than in the other cultivars. The CH7 cultivar exhibited significant enrichment of *norank_f_Entotheonellaceae* and *Dactylosporangium*, and the JX10 cultivar displayed maximum enrichment of *Parafrigoribacterium*. With respect to fungi, the CH12 cultivar significantly recruited *Penicillium*, *Ascobolus*, *Dactylonectria*, *Stachybotrys*, *GS37_gen_Incertae_sedis*, and *Keithomyces*; the JX10 and SS908 cultivars presented significant enrichment of *Naganishia*, *Papiliotrema*, and *Gamsia* (Figure 7b).

### 3.6. Relationship Between Environmental Parameters and Microbial Communities

The RDA was conducted to visualize the correlation between the microbial community data matrix of the five cultivars and environmental factors. For bacteria, most of the tested soil chemical factors were significantly positively correlated with the CH7 cultivar. In addition, the CH12 and JX10 cultivars exhibited significant positive correlation with Leaf yield and Frozen shoot rate, respectively (Figure 7c). With respect to fungi, the CH7 and JX10 cultivars displayed trends similar to those of bacteria, whereas the CH12 and H32 cultivars showed significant positive correlations with TK and leaf yield (Figure 7d).

The correlation network analysis was conducted to assess the complexity of the interactions within the microbial taxa. The results revealed a strong difference between the bacterial and fungal communities (Figure 7e,f). The bacteria exhibited a higher edge number than the fungi, along with a higher proportion of negative edges (Table S5), indicating more complex correlations in the bacterial communities than in the fungal communities in the five cultivars.

**Figure 8:**
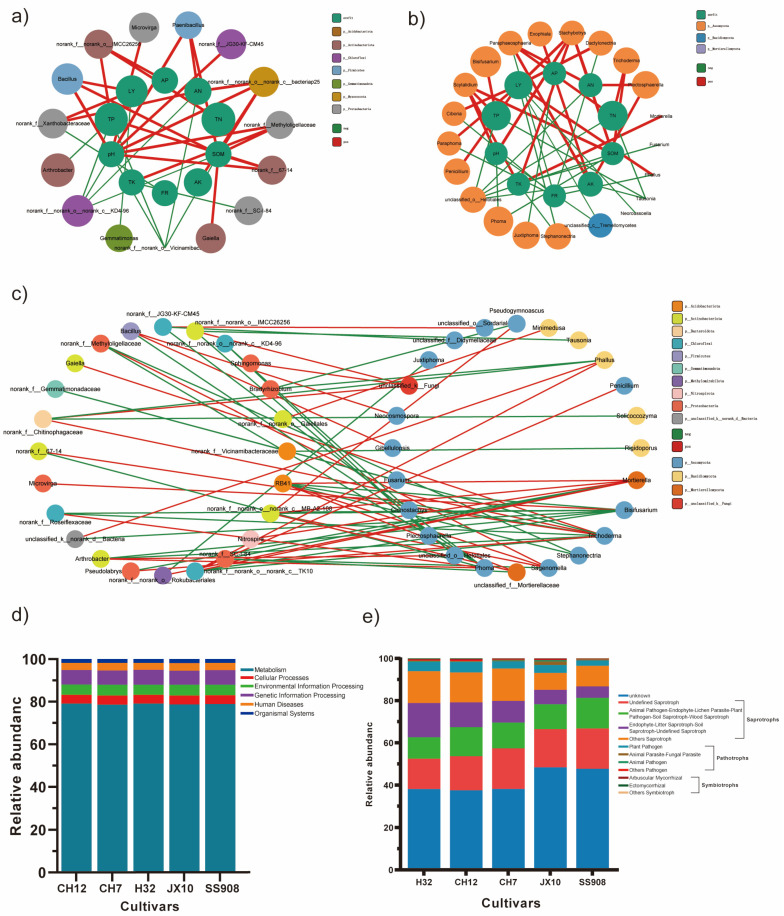
Correlation network analysis of microbial communities. (**a**) Network among bacteria and environment factors. (**b**) Network among fungi and environment factors. (**c**) Network between bacteria and fungi. (**d**) Level 1 of bacteria. (**e**) FUNGuild of fungi.

The correlation network analyses of the microbial communities and soil chemical properties were conducted to study the interactions between them (Figure 8a,b). Proteobacteria and Actinobacteriota were the bacterial phyla most closely related to the soil chemical properties. SOM and pH were most closely related to rhizosphere bacteria, and *g__norank_f__norank_o__norank_c__bacteriap25* was the genus most positively closely related to soil chemical properties. Ascomycota was the fungal phyla most closely related to soil chemical properties. Leaf yield was most closely related to rhizosphere fungi, whereas *Scytalidium* and *Stachybotrys* were the fungal genera most positively closely related to soil chemical properties.

To investigate the potential interactions between bacterial and fungal communities in the mulberry rhizosphere soil, we constructed a correlation network of microbial communities (Figure 8c). Seventy five percent of the bacterial nodes were affiliated with Proteobacteria (25%) and Actinobacteriota (25%), followed by Chloroflexi (16.67%) and Acidobacteriota (8.33%). As for the fungal nodes, 87.5% belonged to Ascomycota (66.67%) and Basidiomycota (20.83). *Trichoderma, Mortierella*, *Bisifusarium*, and *Phoma* were the fungi most closely related to bacteria. *RB41*, *g__norank_f__norank_o__norank_c__MB-A2-108*, and *g__norank_f__norank_o__norank_c__TK10* were the bacteria most closely related to fungi.

### 3.7. Predicted Functional Consequences

The functions of the bacterial communities in all the groups were predicted using six KEGG level 1 functions (metabolism, genetic information processing, environmental information processing, cellular processes, organismal systems, and human diseases) (Figure 8d). Bacteria related to metabolism accounted for over 70%, and there was no significant difference among the different cultivars. FUNGuild was used to analyze fungal function, and the guilds identified in this study are listed in Figure 8e. Over 60% of the sequences in all cultivars were successfully assigned, with a higher guild identification level in the H32, CH12, and CH7 cultivars than in the JX10 and SS908 cultivars. When unassigned OTUs were removed, undefined saprotrophs exhibited the largest guild in OTU richness among all guilds. The second-largest group of guilds included endophyte-litter saprotroph-soil saprotroph-undefined saprotroph in the H32 cultivar, and animal pathogen-endophyte-lichen parasite-plant pathogen-soil saprotroph-wood saprotroph in the other four cultivars (Figure 8e).

## 4. Discussion

The effects of plants on soil functions can be highly species specific, as different plants shape soil functions by uniquely altering soil microbiota, nutrient cycling, and chemical exudation [19]. Soil chemical properties typically vary according to mulberry cultivars [20]. In this study, soil chemical properties differed significantly in different cultivars. The CH7 cultivar was ranked the first and exhibited significantly higher correlation than the other cultivars in all the tested parameters followed by CH12, which indicated the better nutrient uptake and transformation in these two cultivars. Unlike the CH7 cultivar, which has been the highest yielding cultivar in the past, the CH12 cultivar produced significantly higher yields than the other cultivars. This may be attributed to the impaired mulberry function owing to very low winter temperatures and significantly better cold tolerance in the CH12 cultivar than in the other cultivars.

There were significant differences in richness but not in diversity in bacteria and fungi among all the cultivars, which partially agree with the findings of Peng et al. that there are certain differences among cultivars in terms of the diversity and richness of the endophytic community found in mulberry stems [21]. In line with previous studies [22,23], Proteobacteria, Acidobacteriota, and Actinobacteriota were ranked as the top three bacterial phyla with more than 60% of the total bacterial community. Meanwhile, Ascomycota, Basidiomycota, and Mortierellomycota were top three dominant fungal phyla, accounting for more than 80% of the total fungal community. Actinobacteria play a crucial role in soil carbon cycling due to their ability to produce cellulose-degrading enzymes [24], whereas most nitrogen-fixing microorganisms belong to Proteobacteria [25]. Acidobacteria are capable of degrading plant litter in soils [26], Ascomycota is associated with soil nitrogen [27]. Basidiomycota and Mortierellomycota exhibit high respiration rates and SOC mineralization rate [28]. These dominant bacteria and fungi in the rhizosphere were beneficial for carbon and nitrogen cycling in the soil and mulberry trees.

The dominant bacterial genera varied among the cultivars, indicating the complexity of microorganisms under the field experimental conditions. *Bacillus* species are commercially marketed as biofertilizers, biopesticides, and soil amendments with a plant growth promoting effect [29]. Consistently, being one of the dominant bacteria, *Bacillus* was significantly positively associated with SOM and leaf yield in the present study. In addition, *RB41*, *Arthrobacter*, and *Sphingomonas* were the well-known PGPR [30,31,32]. These were the dominant bacteria in all the cultivars used in this study, which may be beneficial for the growth and development of mulberry trees. Furthermore, *norank_f__67-14*, a genus belonging to Actinobacteriota, was reported to be associated with improved pumpkin size [33] and positively correlated with AP [34]. Similarly, we found that *norank_f__67-14* was significantly positively correlated with TP and SOM and not with AP. In addition, *norank_f__Xanthobacteraceae* was closely related to P solubilization and N fixation [35,36,37]. Consistent with previous reports, *norank_f__Xanthobacteraceae* was found to be significantly positively correlated with the AN content and leaf yield in the current study. *Abditibacterium* has been characterized as an oligotrophic chemoheterotrophic bacterium thriving in extreme ecosystems beyond cold ones [38], and showed a preference for soil AP and AK [39]. In the present study, *Abditibacterium* proportion in the CH12 cultivar was significantly higher than in the other cultivars, which may impart strong cold resistance to the CH12 cultivar.

With respect to fungi, *Mortierella* has been reported to increase plant biomass in non-leguminous crop species [40], promote crop growth by increasing biomass, chlorophyll and gibberellic acid [41,42], and desorb phosphate from soil samples by producing oxalic acid [43]. Our findings are partially in agreement with previous reports that *Mortierella*, as being the fourth-most abundant fungus, was significantly positively associated with TK, which favored plant growth. In addition, some genera in this study were significantly positively associated with both leaf yield and soil nutrients, which was consistent with other reports. For instance, *Phallus* was significantly positive with leaf yield, performed multiple functions in soil C, N, and P cycling [44], while also possessing capacity to decompose substrates, allocate, uptake and store nutrients [45]. Although *Ciboria* has been retrieved as plant pathogen causing sclerotiniose in *M. alba* [46] its ability to deplete total petroleum hydrocarbons in the soil in treatment has also been ascertained [47]. *Paraphoma* has been reported to be significantly positively correlated with AN [48], to degrade plant residues and biodegradable plastic [49] and has been shown to have the ability to promote plant growth and secondary metabolite production under abiotic stress [50]. Additionally, some genera in the present study were significantly positively associated with soil nutrients, indirectly benefiting leaf yield, which was also consistent with other reports. For example, *Trichoderma*, which was significantly positively correlated with TN and SOM in the present study, can promote plant growth and participates in bioremediation for the removal of soil contaminants [51,52]. *Plectosphaerella*, which showed a significant positive correlation with AK and AN in this study, has been found to exhibit a significant positive correlation with leaf traits [53] and played a crucial role in organic matter decomposition and nutrient release [54].

In the present study, the relative abundance of *Penicillium*, *Dactylonectria*, and *Stachybotrys* were not only significantly higher in the CH12 cultivar than in the other four cultivars, but also exhibited a positive relation with leaf yield, which contribute to the highest mulberry leaf yield in the CH12 cultivar among all the cultivars. Similarly to our study, many reports demonstrate that *Penicillium* is a key group in P cycling, and is more likely to recruit bacteria that are able to improve its N uptake in addition to P, thereby promoting plant growth [55,56,57,58]. Not only that, but elite strains have also been developed into commercial biofertilizer to improve plant P [55]. *Stachybotrys* acts as cellulose degraders [59], is demonstrated to decompose soil organic matter and promote C, N, and P cycling in the soil [60]. However, unlike our research, *Dactylonectria* has been found to be a key pathogenic fungus negatively correlated with plant growth traits [61], which probably because the strains in the present study were not pathogenic.

It is well known that soil microorganisms do not exist in isolation, but rather co-exist and co-construct a complex network of ecological associations that result in various crucial and complex interactions, including competition, symbiosis, and reciprocity [62]. Therefore, a comprehensive understanding of fungal–bacterial interactions is essential for improving soil conditions [63]. In this study, we found that the interactions among bacteria formed more connections than that those among fungi. Well-connected taxa in co-occurring networks often have a significant impact on the microbial community structure and function, regardless of the abundance across time and space [64]. In our study, the nodes with the highest connections were *RB41* in bacteria with a degree of 6, and *Trichoderma* and *Mortierella* in fungi with a degree of 9 and 7, respectively. This indicated that these genera were relatively active in the interaction network between bacteria and fungi, and whether they have an impact on soil physicochemical properties and plant growth deserves further study.

Instead of being static, plants can actively regulate the assembly of their beneficial microbial communities in response to stress. This dynamic response further highlights the potential to optimize crop yields by exploiting beneficial plant–microbe interactions [65]. Thus, the community assembly plays critical roles in regulating the community, diversity, and functioning of microbes [66]. Liu et al. showed that soybean genotypes can significantly modulate the microbial composition of the rhizobial community and indicated that agricultural breeding programs should consider integrating host traits involved in beneficial microbiome assembly [65]. Our research confirms the assembly differences in rhizosphere microorganisms in mulberry genotypes and supports the view that stochastic processes predominate in the bacterial [67] and fungal community assembly [66]. Unusually, however, the SS908 cultivar was the only cultivar in which deterministic processes predominated during the fungal community assembly, as well as the fact that bacteria were more stochastic than fungi, which need to be further investigated. The higher proportion of generalists and persistent species in bacteria than in fungi indicated more stable bacterial networks. This aligns with the previous finding that fungal communities were more sensitive to *Fusarium wilt* than the bacterial communities [68], but inconsistent with the conclusion that soil bacterial networks were less stable than fungal networks under drought stress [69]. We believe this stems from differences in the environment, species, and soil, among others. Among the five cultivars, the CH12 cultivar, had the highest leaf yield, the most stable bacterial and fungal networks as well as the highest abundance of persistent species. However, the SS908 and JX10 cultivars, which had lower leaf yield, were more sensitive to the environment. Microorganisms exposed to the same environment over an extended period of time leads to the generation of memorized behaviors towards their environment, which helps maintain the relative stability of their community structure, diversity, and function [70]. Plant rhizosphere exudates selectively guide the development of the microbial community structure in a particular direction, thereby establishing mutual memory-like behavior between microorganisms and plants, which in turn influences plant growth [71]. Therefore, whether leaf yield is related to the sensitivity of the rhizosphere microbial community to the environment requires further study. These results indicated that the community assembly processes of bacteria and fungi should not be ignored in regulating interaction between plants and rhizosphere microorganisms.

Fungi are important members of soil microbial communities in crop soils, provide essential ecosystem services, such as nutrient cycling, organic matter decomposition, and soil structure [72]. Fungal taxa were assigned into three ecologically relevant trophic modes-saprotrophy, symbiotrophy, and pathotrophy [73]. Saprotrophic fungi are involved in nutrient cycling, symbiotrophic fungi vastly expand the surface area of plant roots, and pathotrophic fungi attack crop plants but also control other pests [72]. In this study, the saprotrophs occupied over 90% of the assigned OTUs, which was a higher proportion than that reported by Nguyen et al. and Schmidt et al. [72,73]. We believe this is likely related to the differences in the plants tested and the health of the mulberry tree soil used in the experiment.

## 5. Conclusions

In this study, we found that the diversity and assembly processes of rhizosphere bacteria and fungi exhibited significant differences among mulberry cultivars, and their response to soil chemical traits and leaf yield also vary. Proteobacteria, Acidobacteriota, and Actinobacteriota accounted for more than 60% of the total bacterial community, whereas more than 80% of the total fungal community was constituted by Ascomycota, Basidiomycota, and Mortierellomycota. Bacterial and fungal community assembly was dominantly affected by stochastic processes. The most abundant bacterial and fungal species were persistent species. The bacterial community had a higher proportion of generalists and a lower proportion of specialists than the fungal community. During the assembly process of rhizosphere microorganisms in mulberry trees, bacteria were more stable than fungi. The interactions among bacteria formed more connections than that those among fungi. Most tested soil chemical factors were significantly positively correlated with the CH7 cultivar. The CH12 and JX10 cultivars exhibited significant positive correlation with leaf yield and frozen shoot rate, respectively. Soil organic matter and pH were most closely related to rhizosphere bacteria, whereas leaf yield was most closely related to rhizosphere fungi. Collectively, our findings provide insight into the complex relationships among rhizosphere soil microorganisms, mulberry cultivar, and can be used to improve the selection of mulberry varieties, screen beneficial bacteria, and optimize soil nutrient management.

## Figures and Tables

**Table 1:** Soil chemical properties of five cultivars.

	H32	CH12	CH7	JX10	SS908
TN (mg/kg)	883.4 ± 31.2 b	896.5 ± 17.8 b	1375.5 ± 57.4 a	819.8 ± 60.5 b	887.2 ± 53.5 b
TP (mg/kg)	859.8 ± 46.4 b	812.5 ± 53.5 b	1057.3 ± 10.2 a	883.6 ± 4.3 b	834.4 ± 80.3 b
TK (g/kg)	22.5 ± 1 a	20.7 ± 0.4 b	22.6 ± 0.6 a	20.7 ± 0.4 b	20.3 ± 0.5 b
SOM (g/kg)	17.2 ± 2.2 b	18.3 ± 1.6 b	24.9 ± 1.1 a	17.7 ± 1 b	16.2 ± 1.6 b
AN (mg/kg)	110.8 ± 11.1 c	149.8 ± 6 b	167.2 ± 9.4 a	123.1 ± 3 c	117.2 ± 5.7 c
AP (mg/kg)	60.5 ± 5.7 ab	54.3 ± 3 ab	62.6 ± 5.2 a	52.8 ± 2.3 b	57.7 ± 5.2 ab
AK (mg/kg)	210.2 ± 2.6 c	323.6 ± 19.1 ab	376.9 ± 48.8 a	212.7 ± 25.1 c	276.9 ± 49.2 bc
pH	6.9 ± 0.1 b	6.9 ± 0.1 b	7 ± 0.1 a	6.9 ± 0 b	6.7 ± 0.1 c

Note: Data are presented as means ± SD of three replicates. Values within a column followed by different lowercase letters are significantly different (*p* < 0.05).

## Data Availability

The data of this study are openly available upon NCBI. Further inquiries can be directed to the corresponding author.

## Supplementary Materials

The following supporting information can be downloaded at: https://www.mdpi.com/article/10.3390/microorganisms13092157/s1, Table S1. Mulberry planting map. Table S2. PCR details. Table S3. Diversity index of Bacteria. Table S4. Diversity index of Fungus. Figure S1. Rarefaction curves of Bacteria. Figure S2. Rarefaction curves of Fungus. Figure S3. Niche breadth. Figure S4. Niche overlap. Table S5. Correlation network analysis of microbial communities.
