# Supplementary material for: Response of Rhizosphere Microenvironment of Mulberry (Morus alba L.) to Different Cultivars
Source: Microorganisms. 2025 Sep 16;13(9):2157. doi: 10.3390/microorganisms13092157 (PMC12472325; doi:10.3390/microorganisms13092157)
Supplement: Supplementary file 1 [file microorganisms-13-02157-s001.zip › microorganisms-3843256-supplementary.pdf]

Table S1 Mulberry planting map

Line	Mulberry trees				
1	JX10(15plants)	CH7(15plants)	H32(15plants)	CH12(15plants)	SS908(15plants)
2	JX10(15plants)	CH7(15plants)	H32(15plants)	CH12(15plants)	SS908(15plants)
3	JX10(15plants)	CH7(15plants)	H32(15plants)	CH12(15plants)	SS908(15plants)
4	H32(15plants)	CH12(15plants)	SS908(15plants)	JX10(15plants)	CH7(15plants)
5	H32(15plants)	CH12(15plants)	SS908(15plants)	JX10(15plants)	CH7(15plants)
6	H32(15plants)	CH12(15plants)	SS908(15plants)	JX10(15plants)	CH7(15plants)
7	SS908(15plants)	JX10(15plants)	CH7(15plants)	CH12(15plants)	H32(15plants)
8	SS908(15plants)	JX10(15plants)	CH7(15plants)	CH12(15plants)	H32(15plants)
9	SS908(15plants)	JX10(15plants)	CH7(15plants)	CH12(15plants)	H32(15plants)

Table S2 PCR details

	experiment details
PCR mixtures	The PCR mixtures contained 4 μL of 5x TransStartFastPfu buffer, 2 μL of 2.5 mM dNTPs, 0.8 μL of each primer (5 μM each), 0.4 μL of *TransStart*FastPfu DNA Polymerase, 10 ng template DNA, adding ddH_2O to a final volume of 20 μL. All reactions were performed in triplicate.
PCR cycling conditions	PCR cycling conditions included an initial denaturation at 95°C for 3 min, 27 cycles of denaturing at 95°C for 30 s, annealing at 55°C for 30 s, and extension at 72°C for 45 s, followed by a single extension at 72°C for 10 min and a continued hold at 4°C.
Splicing and quality control	Raw sequence reads were demultiplexed, quality-filtered by fastp version 0.20.0, and merged by FLASH version 1.2.7. Operational taxonomic units (OTUs), with a 97% similarity cut-off, were clustered using UPARSE v.7.1, and chimeric sequences were identified and removed. The taxonomy of each OTU representative sequence was analyzed by RDP Classifier v.2.2 against the 16S rRNA database using a confidence threshold of 0.7.

Table S3 Diversity index of Bacteria

	sobs	shannon	simpson	ace	chao	coverage
H32	3247±212a	6.7±0.1a	0.0038±0.0008a	4043±224.6a	3870.2±228.4a	0.974±0.001b
CH12	3186±176a	6.7±0.1a	0.0054±0.0012a	3812.9±202.2ab	3654.4±187.9ab	0.977±0.001ab
CH7	3019±322a	6.7±0.2a	0.0037±0.0007a	3603.1±378.4b	3492.5±370.8b	0.979±0.003a

JX10	3098±73a	6.7±0a	0.0034±0.0002a	3693.1±145.3b	3562±148.7b	0.978±0.002a
SX908	3054±118a	6.7±0a	0.0038±0.0014a	3637±40.9b	3503.4±36.5b	0.979±0.001a

Table S4 Diversity index of Fungus

	sobs	shannon	simpson	ace	chao	coverage
H32	667±10ab	4.1±0a	0.0441±0.0054a	706.7±8.6ab	713.6±12.3ab	0.9987±0a
CH12	669±32ab	4.2±0.1a	0.0337±0.006a	704.6±41.9ab	711.1±44.9ab	0.9989±0.0005a
CH7	622±88b	4.1±0.1a	0.0376±0.0068a	654.1±100.1b	657.7±97.9b	0.9989±0.0005a
JX10	703±81ab	4±0.6a	0.053±0.0325a	777.9±97.3ab	772.1±113.6ab	0.9982±0.0008a
SX908	754±48a	4±0.3a	0.0581±0.0277a	823.4±63a	819.9±56.9a	0.9982±0.0005a

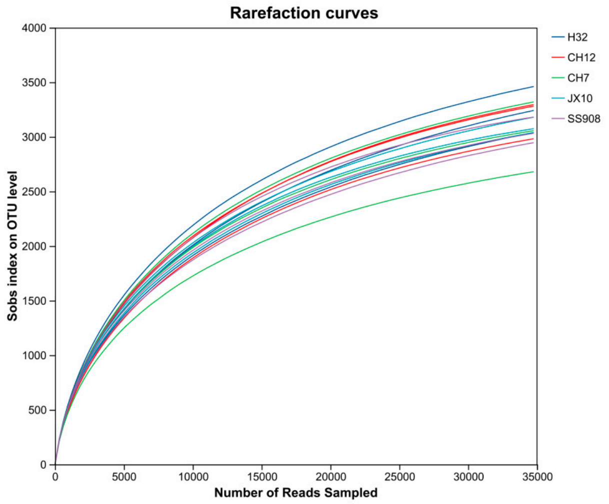

Figure S1 Rarefaction curves of Bacteria

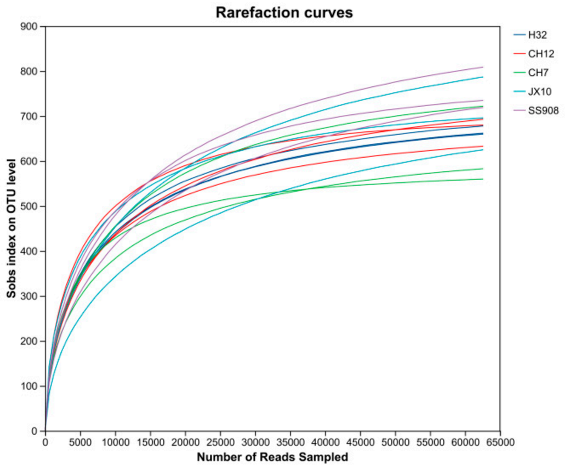

Figure S2 Rarefaction curves of Fungus

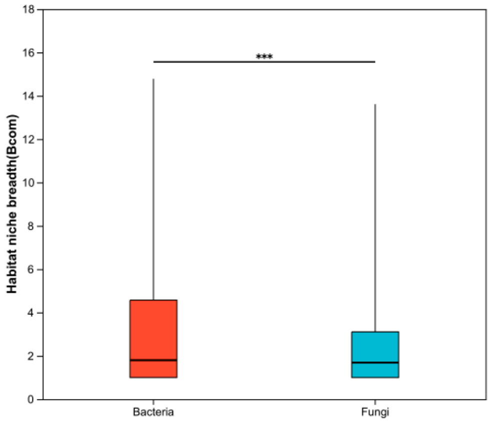

Figure S3 Niche breadth

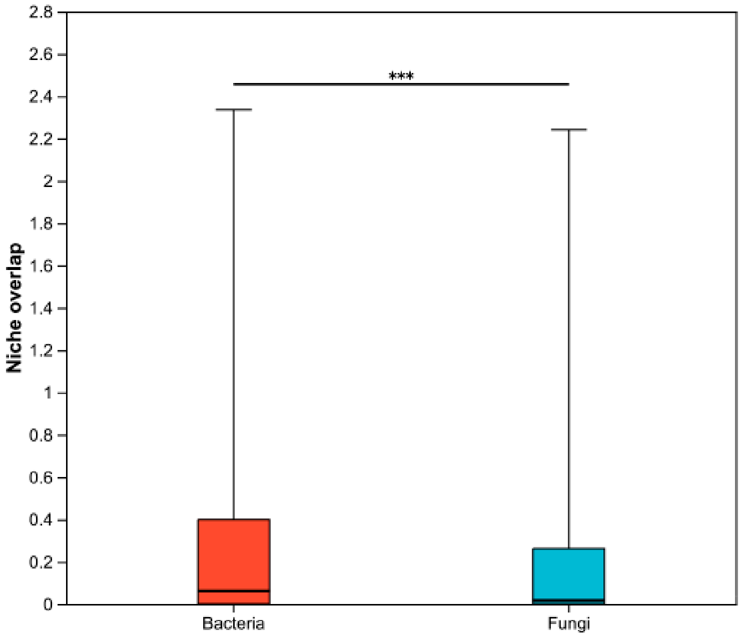

Figure S4 Niche overlap

Tabel S5 Correlation network analysis of microbial communities

Top 50	Node numbers	Edge numbers	Node average degree	Positive edges	Negative edges
Bacteria	49	189	7.71	103	86
Fungi	50	115	4.6	76	39
